# SG-APSIC1048: Lean management dressing set of service unit at Pattani Hospital

**DOI:** 10.1017/ash.2023.115

**Published:** 2023-03-16

**Authors:** Nanthipha Sirijindadirat, Chanitsara Jindarat

**Affiliations:** President, Central Sterilizing Services Association, Bankok, Thailand; Pattani Hospital, Pattani, Thailand

## Abstract

**Background:** Pattani Hospital in southern Thailand has 540 beds and a network of 22 family clinical practice and community locations and 13 primary-care hospitals. We care for patients and soldiers who have wounds from guns and bombs of varying wound size. The central sterilization services department (CSSD) prepares dressing sets with 1 size dressing procedures. **Objectives:** We sought to improve the sizing of dressing sets within Pattani Hospital and network, to reduce preparation time to ≤3 minutes per set, and to reduce the cost of dressing sets by 8,000 Thai baht (US $240) per month. **Methods:** We convened a problem-solving meeting to address the sizes of dressing sets in the hospital. We decided that 3 sizes were needed (small, medium, and large) following the size of the wound. We performed a pilot project and evaluated its progress every 2 months. **Results:** Unique dressing sets were used in 68.18% of cases before the intervention and 88.5% after the sizing changes. Time to prepare the dressing sets decreased from 3 minutes to 1 minute per set. The intervention reduced the costs related to dressing sets from 39,952.50 Thai baht (US $1,194) per month to 25,641 baht (US $766) per month. Furthermore, infectious waste was reduced from 31.8 kg per day to 7.99 kg per day. **Conclusions:** Multiple sizes of dressing sets were prepared for use with wounds of varying size. The CSSD prepared the dressing sets using medical supplies such as gauze, cotton, and top gauze. This project reduced waste and improved cost-effectiveness, and this procedure will be extended to other care facilities in our network.

